# Tools and scores for perioperative pulmonary, renal, hepatobiliary, hematological, and surgical site infection risk assessment: an update

**DOI:** 10.1590/0100-6991e-20223125-en

**Published:** 2022-07-08

**Authors:** CAIO MAZZONETTO TEÓFILO DE MORAES, LUISA DE MENDONÇA CORRÊA, RICARDO JAYME PROCÓPIO, GABRIEL ASSIS LOPES DO CARMO, TULIO PINHO NAVARRO

**Affiliations:** 1 - Universidade Federal de Minas Gerais - Belo Horizonte - MG - Brasil; 2 - Faculdade Ciências Médicas de Minas Gerais - Belo Horizonte - MG -Brasil; 3 - Universidade Federal de Minas Gerais, Hospital das Clínicas, Unidade Endovascular - Belo Horizonte - MG - Brasil; 4 - Universidade Federal de Minas Gerais, Departamento de Clínica Médica - Belo Horizonte - MG - Brasil; 5 - Universidade Federal de Minas Gerais, Departamento de Cirurgia - Belo Horizonte - MG - Brasil

**Keywords:** Perioperative Period, Risk Assessment, Postoperative Complications, Decision Support Techniques, General Surgery, Sistemas de Apoio a Decisões Clínicas, Período Perioperatório, Complicações Intraoperatórias, Complicações Pós-Operatórias, Cirurgia Geral

## Abstract

**Introduction:**

perioperative risk assessment is essential to mitigate surgical complications, which suggests individual and collective interest since the number of surgical procedures in Brazil has been expanding steadily. The aim of this study was to summarize and detail the main calculators, indexes and scores regarding perioperative pulmonary, renal, hepatobiliary, hematological and surgical site infection risks for general non-cardiac surgeries, which are dispersed in the literature.

**Method:**

a narrative review was performed based on manuscripts in English and Portuguese found in the electronic databases Pubmed/MEDLINE and EMBASE.

**Results:**

the review included 11 tools related to the systems covered, for which the application method and its limitations are detailed.

**Conclusion:**

the non-cardiovascular perioperative risk estimation tools are beneficial when disturbances are identified in the preoperative clinical examination that justify a possible increased risk to the affected system, so the use of these tools provides palpable values to aid in the judgment of surgical risk and benefit as well as it identifies factors amenable to intervention to improve outcomes.

## RESUMO

**Introdução:**

a avaliação de risco perioperatório é essencial para mitigação das complicações cirúrgicas, o que aventa interesse individual e coletivo uma vez que o número de procedimentos cirúrgicos no Brasil vem se expandindo de maneira crescente. O objetivo deste estudo foi resumir e detalhar as principais calculadoras, índices e escores dos riscos perioperatórios pulmonar, renal, hepatobiliar, hematológico e de infecção de sítio cirúrgico para cirurgias gerais não cardíacas, os quais encontram-se dispersos na literatura.

**Método:**

foi realizada revisão narrativa a partir de manuscritos em inglês e português encontrados nas bases eletrônicas Pubmed/MEDLINE e EMBASE.

**Resultados:**

a revisão incluiu 11 ferramentas relativas aos sistemas abordados, para as quais detalha-se o método de aplicação e suas limitações.

**Conclusão:**

as ferramentas de estimativa de risco perioperatório não cardiovascular encontram benefício quando se identifica no exame clínico pré-operatório alterações que justifiquem possível risco aumentado ao sistema afetado, assim a utilização destas ferramentas fornece valores palpáveis para auxílio no julgamento de risco e benefício cirúrgico bem como identifica fatores passíveis de intervenção para melhoria dos desfechos.

## INTRODUCTION

Perioperative risk assessment invariably begins with anamnesis and physical examination of the patient for whom a surgical intervention is considered. From this moment on, the evaluating physician judges the need of obtaining additional data, which will help in the joint decision with the patient and family, weighing risk and benefit, as well as interventions for preoperative clinical stabilization.

Among the mechanisms for obtaining data for the objective assessment of the patient are the perioperative risk indexes, scores, and calculators, which complement the physician’s initial assessment. Tools for general and cardiovascular risk - for example, the American Society of Anesthesiologists (ASA)^1^ classification and the Revised Cardiac Risk Index (RCRI)^2^, respectively - receive emphasis in the preoperative period assessment. However, the risk of complications in other organ systems must be suspected and can also benefit from the estimation promoted by similar tools.

The structuring of the perioperative risk assessment proved to be fruitful in view of the large number of surgical procedures performed worldwide^3^ and, in the Brazilian context, the accelerated growth^4^, with room for expansion due to the high demand not yet met^5^. Nonetheless, information on perioperative risk estimation tools is dispersed in the literature. This can be a problem for the physician who assesses the patient preoperatively, especially for perioperative risk of non-cardiovascular complications. Therefore, gathering, organizing, and detailing such information equips the reader with the critical eye necessary for choosing the appropriate tool.

This article aims to address the dispersion of non-cardiovascular perioperative risk assessment tools by synthesizing the main calculators, indices, and scores regarding perioperative pulmonary, renal, hepatobiliary, hematological, and surgical site infection risks for general, non-cardiac surgeries.

## METHODS

This is a narrative review carried out by searching the Pubmed/MEDLINE and EMBASE electronic databases for manuscripts in English and Portuguese. We chose this method because it has the advantage of allowing the aggregation of different elements - that is, the risk assessment tools for several systems - within a single text. However, we should note that the format is susceptible to subjectivity.

### Pulmonary Risk

The assessment of the risk of pulmonary complications for a long time remained undervalued to the detriment of cardiac risk in surgeries. This has changed in recent decades, since it has been identified that pulmonary complications can occur with a frequency similar to cardiac complications and determine a longer hospital stay, in addition to both often occurring concomitantly^6^ ^-^ ^8^.

Perioperative pulmonary complications can be of different nature^8^ ^-^ ^10^, but the most clinically relevant are atelectasis, pneumonia, respiratory failure, and exacerbation of underlying chronic lung disease^11^. For these perioperative outcomes, multiple risk factors have already been demonstrated, namely, type of surgery, advanced age (over 60 years), chronic obstructive pulmonary disease, smoking, heart failure, functional dependence, ASA classification, obesity, impaired consciousness (confusion, delirium, but not dementia or chronic mental illness), abnormal findings on chest examination, alcohol use, and weight loss^9^ ^,^ ^11^ ^,^ ^12^.

Several indices and risk calculators for perioperative complications in this system have been developed. They differ in the variables considered, outcomes, and populations studied and no tool is suitable for all situations^13^ ^-^ ^20^. Due to their good clinical applicability and risk estimation capability, we highlight the Respiratory Failure Risk Index^18^, the Postoperative Pneumonia Risk Index^19^, and the Assess Respiratory Risk in Surgical Patients in Catalonia (ARISCAT)^20^, described below.

The Respiratory Failure Risk Index was proposed in 2000, developed from a prospective cohort of 81,719 patients undergoing noncardiac surgery, and validated on a set of 99,390 patients. The outcome was postoperative respiratory failure, defined by use of mechanical ventilation for more than 48 hours after surgery or reintubation with mechanical ventilation after extubation. This outcome is relevant insofar as, although uncommon, it incurs a substantial increase in 30-day mortality - in this study, 27% of mortality occurred in patients who had postoperative respiratory failure and 1% in those who did not. The main limitations of the study were the exclusion of female patients, a population with a high prevalence of comorbidities, and the absence of pulmonary function tests prior to surgery. The index is performed by scoring each risk factor, and the sum categorizes the patient into one of five classes with increasing risk of postoperative respiratory failure, as seen in Tables 1 and 2^18^. Although the index considers the score of only some types of surgery as risk factors for respiratory failure, all types of non-cardiac surgery performed under general, spinal, or epidural anesthesia were included in its development, except for transplants and procedures with very low mortality, such as dental procedures, endoscopy, and central venous catheter insertion.

**Table 1:** Risk factors and postoperative Respiratory Failure Risk Index scores ^18^ .

Risk factors	Score
Type of surgery	
Abdominal aortic aneurysm	27
Thoracic	21
Neurosurgery, upper abdominal, or peripheral vascular	14
Neck	11
Emergency surgery	11
Albumin <30g/L	8
Urea >62mg/dL	8
Total or partial functional dependence	7
History of chronic obstructive pulmonary disease	6
Risk factors	Score
Age	
≥70 years	6
60-69 years	4

**Table 2:** Classes, scoring and estimated risk by postoperative Respiratory Failure Risk Index ^18^ .

Class	Score	Risk of postoperative respiratory failure (%)
I	≤10	0.5
II	11-19	2.2
III	20-27	5
IV	28-40	11.6
V	>40	30.5

The Postoperative Pneumonia Risk Index was published in 2001, developed from medical records of 160,805 patients, and validated by data from 155,266 individuals, all of whom underwent noncardiac surgery. The outcome is postoperative pneumonia, which is relevant due to the increase in 30-day mortality - in the study, patients with postoperative pneumonia had a 21% mortality rate versus 2% in patients without it. Among its main limitations is the population with a high prevalence of comorbidities and low participation of female patients (3.2%), which may limit its usefulness for more heterogeneous and healthy populations. In addition, the list of risk factors that score in the index is lengthy, which can make its use difficult. All surgeries in which there was general, epidural, spinal, or local anesthesia were included, excluding only transplants and surgeries with very low mortality. Possibly the small proportion of participants undergoing orthopedic procedures may have underestimated the risk of this type of surgery. The index is determined by scoring risk factors and categorizing the patient into 5 classes (Tables 3 and 4)^19^.

**Table 3:** Risk factors and scores for the Postoperative Pneumonia Risk Index ^19^ .

Risk factors	Score
Type of surgery	
Abdominal aortic aneurysm repair	15
Thoracic	14
Risk factors	Score
Upper abdominal	10
Neck	8
Neurosurgery	8
Vascular	3
Age	
≥80 years	17
70-79 years	13
60-69 years	9
50-59 years	4
Functional status	
Totally dependent	10
Partially dependent	6
Weight loss >10% in the last 6 months	7
History of chronic obstructive pulmonary disease	5
General anesthesia	4
Impaired Sensory	4
History of stroke	4
Urea	
<17mg/dL	4
47-64mg/dL	2
≥64mg/dl	3
Transfusion >4 units of packed blood cells	3
Emergency surgery	3
Chronic steroid use	3
Active smoker (in the last year)	3
Alcohol consumption >2 drinks/day in the prior 2 weeks	2

**Table 4:** Classes , scoring and estimated risk by the Postoperative Pneumonia Risk Index ^19^ .

Class	Score	Risk of postoperative pneumonia (%)
I	0-15	0.2
II	16-25	1.2
III	26-40	4
IV	41-55	9.4
V	> 55	15.3

The Assess Respiratory Risk in Surgical Patients in Catalonia (ARISCAT), published in 2010, developed from a prospective multicenter study a postoperative pulmonary risk index that, unlike the two previous indices, identifies multiple outcomes: respiratory infection, respiratory failure, bronchospasm, atelectasis, pleural effusion, pneumothorax, and aspiration pneumonitis. The relevance of these complications is demonstrated by the 20% mortality of patients with postoperative pulmonary complications in the sample studied. The substantially smaller sample (2,464 patients) than the previous indices and the development from a specific population in Spain (Catalonia) may limit its application in more heterogeneous populations. The construction of a patient’s risk level is based on the sum of the scores obtained from seven different risk factors, classifying as low, moderate, or high risk of complications, as shown in Tables 5 and 6^20^.

**Table 5:** Postoperative pulmonary risk factors and scores according to the ARISCAT index ^20^ .

Risk factors	Score
Age	
51-80	3
>80	16
Preoperative PaO2 (%)	
91-95	8
≤90	24
Respiratory tract infection in the last month	17
Preoperative anemia (Hb ≤10g/dL)	11
Surgical incision	
Upper abdominal	15
Intrathoracic	24
Surgery duration (hours)	
2-3	16
>3	23
Emergency procedure	8

ARISCAT: Assess Respiratory Risk in Surgical Patients in Catalonia.

**Table 6:** Risk levels of pulmonary complications estimated by ARISCAT ^20^ .

Risk level	Score	Risk of postoperative pulmonary complications (%)
Low	<26	0.7 - 1.6
Moderate	26-44	6.3 - 13.3
High	≥45	42.1 - 44.9

ARISCAT: Assess Respiratory Risk in Surgical Patients in Catalonia.

### Kidney risk

Renal dysfunction, generally measured by serum creatinine and estimated glomerular filtration rate, increases perioperative complications and mortality, especially cardiovascular complications^21^ ^,^ ^22^. The use of dialysis^23^ and the presence of acute renal failure^24^ also contribute to an increased risk of perioperative complications.

Acute kidney injury (AKI) is not uncommon in the perioperative period. It occurs in around 12% of major elective surgeries, ranging from 1% to 18.7% depending on the type of procedure, patient risk factors, and perioperative management^25^ ^-^ ^29^. Postoperative AKI contributes substantially to the increase in hospital expenses^30^ and, even when mild, constitutes a risk factor for prolonged stay in the intensive care unit (ICU)^25^. Renal risk indices that have been proposed for general non-cardiac surgery focus on predicting the probability of postoperative AKI^29^ ^,^ ^31^.

We highlight two of these tools here. The General Surgery Acute Kidney Injury Risk Index was proposed in 2009, based on data from 75,952 surgeries in the United States, found 11 independent risk predictors (grouped into nine items), and estimated the risk in five classes, as seen in Table 7 ^29^. Among its limitations are the exclusion of vascular, urological, ophthalmological, obstetric, and cardiac procedures, and the lack of data on intraoperative hydration.

**Table 7:** Risk Index for Acute Kidney Injury in General Surgery ^29^

Independent risk factors	Class (risk factors present)	Risk of AKI (%)
Age ≥56 years	I (0-2)	0.2
Male	II (3)	0.8
Cardiac insufficiency	III (4)	1.8-2
Ascites	IV (5)	3.3-3.6
Hypertension	V (6 or more)	8.9-9.5
Emergency surgery		
Intraperitoneal surgery		
Mild or moderate renal impairment*		
Diabetes mellitus under treatment**		

*preoperative serum creatinine >1.2mg/dL; **oral or insulin treatment; AKI: acute kidney injury.

In 2019, the Simple Postoperative AKI Risk (SPARK) was published, based on data from 79,518 patients, seeking to predict AKI. The SPARK uses a score according to Tables 8 and 9 and is suggested for surgeries in which the patient is stable and there is no specific risk that requires a more detailed assessment (nephrectomy, for example)^31^. This tool is limited to include only general, orthopedic, gynecological, obstetric, urological, and neurosurgical procedures, with reduced performance in the last two. It was also developed in an Asian population, in South Korea, which may be a limitation when applied to more heterogeneous populations.

**Table 8:** Preoperative risk factors for renal risk and their SPARK scores ^31^ .

Risk factor	Score	Risk factor	Score
Age years)		Estimated GFR (mL/min/1.73m²)	
<40	0	≥60	0
≥40 and <60	6	≥45 and <60	8
≥60 and <80	9	≥30 and <45	15
≥80	13	≥15 and <30	22
Emergency surgery	7	Albuminuria on the tape test	6
Expected duration of surgery (hours)	5	Diabetes mellitus	4
Sex		Use of RAAS blocker*	6
Male	0	Hypoalbuminemia (<3.5g/dL)	8
Female	8	Anemia*	4
		Hyponatremia (<135mEq/L)	3

*RAAS: renin-angiotensin-aldosterone system; Anemia: serum hemoglobin <12g/dL for women and <13g/dL for men; GFR: glomerular filtration rate; SPARK: Simple Postoperative AKI Risk.

**Table 9:** Classes and estimated Renal risk according to SPARK Index ^31^ .

SPARK Class	Score	Risk of AKI (%)	Critical AKI risk (%)
A	<20	<2	<2
B	20-39	≥2	<2
C	40-59	≥10	≥2
D	≥60	≥20	≥10

SPARK: Simple Postoperative AKI Risk; AKI: acute kidney injury.

Both the General Surgery AKI Risk Index and the SPARK excluded patients with previous chronic kidney disease and did not consider the perioperative use of nephrotoxic agents.

### Hepatobiliary risk

The literature on perioperative hepatobiliary risk estimation focuses on the identification of increased risk of morbidity and mortality in cirrhotic patients^32^ ^-^ ^40^, given that the safety of elective surgeries in patients with mild chronic liver disease has already been reported^41^ ^,^ ^42^. Acute hepatitis substantially increases the perioperative risk, such that in these cases clinical treatment is recommended, and surgery is postponed whenever possible^43^ ^-^ ^45^. In cirrhotic patients, perioperative mortality is around 7-9% in elective surgeries^33^ ^,^ ^38^. Two tools initially developed for different purposes were successfully adapted to estimate the risk of perioperative mortality in cirrhotic patients: the Child-Turcotte-Pugh score and the Model for End-stage Liver Disease (MELD).

The Child-Turcotte-Pugh (CTP) score was initially used to identify the level of liver dysfunction and the severity of portal hypertension in cirrhotic patients^46^. Despite being old (1973), the score started to be used for risk estimation and remained with good accuracy over time^44^. It is composed of five stratified parameters, to which a score is assigned, as shown in Tables 10 and 11. Among its limitations, the CTP score has the possible variation between evaluators (in the encephalopathy and ascites items) and the small sample in the studies that used it as a risk estimation method. Studies that identify the CTP score as a risk estimation model or as a factor associated with increasing mortality according to patient classification include different types of surgery: general abdominal surgery^33^ ^,^ ^37^, neurosurgery, head and neck, ophthalmologic, facial, thoracic, vascular, urological, and gynecological surgery^35^.

**Table 10:** Parameters for Hepatobiliary risk according to the Child-Turcotte-Pugh score ^46^ .

Parameters	Points		
	1	2	3
Encephalopathy	Absent	Grade I or II	Grade III or IV
Ascites	Absent	Mild	Moderate
Total bilirubin (mg/dL)	<2	2-3	>3
Albumin (g/L)	>3.5	2.8-3.5	<2.8
Prothrombin time (extended seconds)	<4	4-6	>6

**Table 11:** Classes ^46^ and perioperative mortality due to hepatobiliary risk according to the Child-Turcotte-Pugh score ^33^ ^,^ ^37^ .

Class	Score	Mortality (%)
A	5-6	10
B	7-9	17-30
C	10-15	63-82

MELD was proposed in 2000 with the aim of predicting mortality after transjugular intrahepatic portosystemic shunt (TIPS)^47^. It is calculated by the following formula, approximating the result to the nearest whole number: MELD = 3.78 x ln(serum bilirubin in mg/dL) + 11.2 x ln(INR) + 9.57 x ln(serum creatinine in mg/dL) + 6.43. INR is the International Normalized Ratio obtained from the prothrombin time. The literature indicates that the perioperative 30-day mortality predicted by MELD ranges from 5.7%, with MELD <8, to 54%, when >15^33^ ^,^ ^36^ ^,^ ^38^. The guidelines of the American Society of Gastroenterology define that MELD <16 expresses a reduced risk in relation to higher values^48^, and MELD is associated with long-term postoperative mortality^36^. Among the types of surgeries considered to identify the relationship between MELD and perioperative mortality are general abdominal^33^ ^,^ ^36^ ^,^ ^38^, orthopedic, and cardiovascular^36^ surgeries.

### Hematological and thromboembolic risk

In the hematological evaluation of the pre-surgical patient, one mainly seeks to identify anemia and coagulation disorders. Anemia is directly clarified by the blood count and other complementary tests, without the application of specific risk scores. A structured clinical history is usually sufficient to exclude the risk of increased perioperative bleeding without the need for additional tests^49^. Thus, the main hematological condition that benefits from risk stratification by scores is venous thromboembolism (VTE).

The incidence of VTE (deep vein thrombosis - DVT - and pulmonary thromboembolism - PTE) has had little variation over the years despite the evolution in treatment and prophylaxis^50^ ^,^ ^51^. Its occurrence substantially increases the costs of hospitalized patients^51^, and it is the leading cause of preventable in-hospital death^52^ ^,^ ^53^. The prevalence in hospitalized or recently hospitalized individuals is 0.8-1.2%^52^ ^-^ ^55^. Surgical patients are at increased risk for VTE^52^ ^,^ ^56^, especially if undergoing high-risk^57^ or long^55^ procedures. There are several models of VTE risk assessment, among which the most used is the Caprini score, which is highlighted by the guideline for VTE prevention in non-orthopedic surgical patients of the American College of Chest Physicians - ACCP) with the Rogers method^58^.

The Caprini score was originally published in 2005^59^. It considered VTE up to 30 days after surgery and is based on the cumulative sum of the patient’s risk factors, having received external validation in several studies for different types of procedures, including general, vascular, urological^60^, plastic or reconstructive^61^, orthopedic, and transplant surgery in critically ill patients^62^. The score underwent adaptations and Table 12 shows the proposal by the ACCP. Patients are categorized into very low (0-1 point), low (2 points), moderate (3-4 points), or high (≥5 points) risk, corresponding to the estimate of VTE occurrence of < 0.5%, 1.5%, 3%, and 6%, respectively^58^. The risk of VTE increases significantly in patients with a Caprini score ≥8, these being the patients who benefit most from chemoprophylaxis^61^ ^-^ ^63^.

**Table 12:** Risk factors for venous thromboembolism according to the modified Caprini score (ACCP) ^58^ .

1 point	2 points	3 points	5 points
Age 41-60 years	Age 61-74 years	Age ≥75 years	Stroke (<1 month)
Minor surgery	Arthroscopy surgery	VTE history	Elective arthroplasty
BMI >25kg/m²	Major open surgery (>45 min)	VTE family history	Fracture of hip, pelvis, or leg
Lower limb edema	Laparoscopic surgery (45 min)	Factor V of Leiden	Acute spinal cord injury (<1 month)
Varicose veins	Malignancy	Prothrombin 202010A	
Pregnancy or puerperium	Restricted to bed (>72h)	Lupus anticoagulant	
History of recurrent or unexplained miscarriage	Immobilized or plastered	Anticardiolipin antibody	
OAC use or hormone replacement	Central venous access	Elevated serum homocysteine	
Sepsis (<1 month)		Heparin-induced thrombocytopenia	
Major lung disease, including pneumonia (<1 month)		Other congenital or acquired thrombophilia	
Altered lung function			
Acute myocardial infarction			
Heart failure (<1 month)			
History of inflammatory bowel disease			
Bedridden patient			

OAC: oral contraceptive; BMI: body mass index; VTE: venous thromboembolism; ACCP: American College of Chest Physicians.

The Rogers score (Patient Safety in Surgery Venous Thromboembolism Score) was proposed in 2007 from a sample of 183,069 patients in which independent variables were identified that were associated with increased risk of postoperative VTE. The index was developed including abdominal, musculoskeletal, thoracic, vascular, and head and neck surgeries (excluding urologic, gynecologic, ophthalmologic, neurosurgery, and auditory tract surgery) and it is calculated by adding the values assigned to the patient’s risk factors, as shown in Table 13. This sum will classify the patient at low, medium, or high risk of VTE, as seen in Table 14 ^64^. Despite being a well-structured formulation, this tool has a lengthy application and lacks external validation^58^.

**Table 13:** Risk factors for venous thromboembolism according to the Rogers score ^64^ .

Risk factor	Punctuation
Type of surgery (non-endocrinological)	
Respiratory or blood	9
Thoracoabdominal aneurysm, embolectomy/thrombectomy, venous reconstruction, endovascular repair	7
Aneurysm	4
Mouth, palate	4
Stomach, intestines	4
Skin	3
Hernia	2
ASA rating	
3, 4 or 5	2
2	1
Female	1
Relative unit of work (%)	
>17	3
10-17	2
Widespread cancer	2
Chemotherapy for malignancy within 30 days after surgery	2
Preoperative sodium >145mmol/L	2
Transfusion >4 units of PBC up to 72h before surgery	2
Ventilator dependence	2
Potentially contaminated wound	1
Preoperative hematocrit ≤38%	1
Preoperative bilirubin >1.0mg/dL	1
Dyspnea	1
Albumin ≤3.5mg/dL	1
Emergency	1

ASA: American Society of Anesthesiologists; Relative Unit of Work (Work RVU): a unit of work determined by the US Department of Health and Human Services, Centers for Medicaid and Medicare; PBC: packed blood cells.

**Table 14:** Risk levels for venous thromboembolism according to the Rogers score ^64^ .

Risk level	Score	Estimated VTE risk (%)
Low	<7	0.1
Moderate	7-10	0.5
High	>10	1.37

### Surgical site infection

Surgical site infections (SSI) represent an important fraction of nosocomial infections in surgical patients and may be responsible for 38% of these infections depending on the scenario analyzed, as exposed by Malone et al. in a study with 6,301 North American individuals, mostly male (95%), undergoing non-cardiac surgery^65^. SSI occur in 1.2-3.9% of surgeries^65^ ^-^ ^67^, which may vary depending on external factors such as adequacy of the antibiotic prophylaxis protocol^68^ ^-^ ^70^, surgeon’s experience in specific procedures^71^, and operation site, with a tendency towards a higher incidence in developing countries^72^.

A well-established and old way of classifying operative wounds was developed by the US National Research Council in 1964, grading into clean, potentially contaminated, contaminated, and infected. Despite widely known, this traditional wound classification system has limited accuracy in risk estimation, especially as it does not consider factors intrinsic to the patient^73^. Thus, some tools for SSI risk estimation were developed.

In 1985, an American study with 58,498 patients was published proposing a risk index for surgical site infection, as part of the Study on the Efficacy of Nosocomial Infection Control (SENIC) project. This index estimates the risk of superficial or deep SSI based on four risk factors, as shown in Table 15. Each risk factor correspond to 1 point to the index and patients with 0 points are considered low risk, patients with 1 point, moderate risk, and high risk with 2 points or more. The study states that the index is capable of adequately predicting 90% of SSI cases, with greater precision than the traditional wound classification system^74^.

**Table 15:** Surgical site infection risk index of the SENIC project, 1985 ^74^ .

Risk factors (one point each)	Score	Surgical site infection (%)
Abdominal surgery	0	1
Surgery duration >2 hours	1	3.6
Contaminated or infected surgery*	2	8.9
Patient has ≥3 diagnoses	3	17.2
	4	27

SENIC: Study on the Efficacy of Nosocomial Infection Control ; *according to the traditional wound classification.

In 1991, an adaptation to the SENIC project index was proposed, also in the United States: the Surgical Site Infection Risk Index, based on the National Nosocomial Infections Surveillance (NNIS) program. In this index, like the previous one, each risk factor corresponds to 1 point to the patient’s total score. The factors considered are: 1) the ASA rating (1 point if ≥3); 2) wound classification by the traditional system (1 point if contaminated or infected) and; 3) duration of surgery (1 point if above the 75^th^ percentile - the study provides a table with cut-off times for each type of procedure, in hours). For patients with 0, 1, 2, and 3 points, the incidence of SSI estimated by this tool is 1.5%, 2.9%, 6.8%, and 13%, respectively^73^.

More recently, in 2013, the Surgical Site Infection Risk Score (SSIRS)^67^ was published, based on a derivation and validation samples from approximately 180,000 patients. This model estimates the risk of superficial or deep surgical site infection up to 30 days after the procedure. The risk is estimated through a calculator considering the characteristics of the patient and the surgery, available in English at: http://www.ohri.ca/SSI_risk_index/Default.aspx. The calculator asks for information on smoking (yes or no), weight, height, medical history (peripheral vascular disease, metastatic cancer, use of corticosteroids for at least 10 days, systemic inflammatory response syndrome, or sepsis in the last 2 days), and surgery (inpatient or outpatient, emergency or not, wound classification, ASA class^1^, general anesthesia or not, additional procedure, surgery time, and type of surgery).

## FINAL CONSIDERATIONS

Preoperative risk assessment is usually focused on the cardiovascular system, may benefit from extending the analysis to other systems. In practice, these other systems’ risk estimation tools will be beneficial in patients in whom, by clinical examination, alterations are identified that raise the possibility of increased risk. For example, in cirrhotic patients, the help of the CTP^46^ or MELD^47^ tools is of interest insofar as they provide tangible values for surgical decision making, considering risk and benefit.

The identification of factors considered in the composition of scores and calculators can also guide perioperative interventions aimed at risk reduction. For example, smoking is one of the factors that comprise the calculation of the risk of surgical site infection according to SSIRS^67^. Therefore, in elective surgeries, previous smoking cessation, in addition to the known systemic benefits, may reduce the incidence of surgical site infection.

This way, the physician who evaluates the patient in the preoperative period can choose to use the most appropriate tool, as deemed necessary, considering its method of application herein described, and its advantages and limitations, as also presented throughout this work and summarized in Table 16. Great attention should be paid to the surgical specialties that were excluded from the development of each tool to know the applicability of the risk estimation method for the type of surgery to be performed.

**Table 16:** Main advantages and limitations of risk assessment tools by system.

Tool (year of publication)	Benefits	Limitations
Pulmonary risk		
Respiratory Failure Risk Index (2000)^18^	Simple application (sum of points). Considers type of surgery.	Only included male patients. Evaluates single outcome
Postoperative Pneumonia Risk Index (2001)^19^	Simple application (sum of points). Considers type of surgery.	Validated for population with high prevalence of comorbidities and low female participation. Evaluates single outcome
ARISCAT (2010)^20^	Simple application (sum of points). Evaluates multiple outcomes	Relatively small sample. Validated for specific population (Catalonia)
Kidney risk		
AKI Risk Index in General Surgery (2009)^29^	Simple application (sum of points). Considers the operated site.	Vascular, urological, ophthalmological, and obstetric procedures excluded. Did not evaluate the use of nephrotoxic agents and intraoperative hydration.
Tool (year of publication)	Benefits	Limitations
Simple Risk of Postoperative AKI - SPARK (2019)^31^	Simple application (sum of points). Uses several factors that can be preoperatively adjusted. Includes obstetric procedures	Reduced performance in urological and neurosurgical procedures. Did not evaluate the use of nephrotoxic agents. Developed in a specific population (South Korea).
Hepatobiliary Risk		
Child-Turcotte-Pugh Score - CTP (1973)^46^	Simple application (sum of points).	Possible variation between evaluators (degrees of ascites and encephalopathy).
Model for End-Stage Liver Disease - MELD (2000)^47^	Quick application (requires calculator). Uses only 3 variables.	Few types of surgery (abdominal, orthopedic, cardiovascular)
Hematological and thromboembolic risk		
Caprini (2005)^59^	Cut-off point indicating chemoprophylaxis for VTE. Considers the type of surgery.	Lengthy application (37 items to consider)
Rogers (2007)^64^	Considers the type of surgery.	Lengthy application (23 items to consider).
Surgical site infection		
SENIC (1985)^74^	Simple application (sum of points).	Old, developed from hospital records from the years 1970-1976 in the United States.
SSIRS (2013)^67^	Simple application (digital calculator). Targets the specific type of surgery to be performed, including all specialties.	Risk in unclean wounds can be underestimated by the use of antibiotics. Of the 14 factors considered, only 2 are potentially modifiable (smoking and surgery time)

ARISCAT: Assess Respiratory Risk in Surgical Patients in Catalonia; AKI: acute kidney injury; SPARK: Simple Postoperative AKI Risk; MELD: Model for End-stage Liver Disease; VTE: thromboembolism venous; SENIC: Study on the Efficacy of Nosocomial Infection Control; SSIRS: Surgical Site Infection Risk Score.

One of the limitations of this study is the subjectivity inherent to the narrative review model. The model was chosen because it allows the aggregation of different topics about the use of tools for non-cardiac perioperative risk assessment in a single text. However, it is impossible to exclude some subjectivity in the selection and interpretation of the bibliography contained herein.

## References

[1] Saklad M (1941). Grading of patients for surgical procedures. Anesthesiology.

[2] Lee TH, Marcantonio ER, Mangione CM, Thomas EJ, Polanczyk CA, Cook EF (1999). Derivation and Prospective Validation of a Simple Index for Prediction of Cardiac Risk of Major Noncardiac Surgery. Circulation.

[3] Weiser TG, Regenbogen SE, Thompson KD, Haynes AB, Lipsitz SR, Berry WR (2008). An estimation of the global volume of surgery a modelling strategy based on available data. Lancet.

[4] Yu PC, Calderaro D, Gualandro DM, Marques AC, Pastana AF, Prandini JC (2010). Non-Cardiac Surgery in Developing Countries Epidemiological Aspects and Economical Opportunities - The Case of Brazil. PLoS ONE.

[5] Rose J, Weiser TG, Hider P, Wilson L, Gruen RL, Bickler SW (2015). Estimated need for surgery worldwide based on prevalence of diseases a modelling strategy for the WHO Global Health Estimate. Lancet Glob Health.

[6] Lawrence VA, Dhanda R, Hilsenbeck SG, Page CP (1996). Risk of Pulmonary Complications After Elective Abdominal Surgery. Chest.

[7] Smetana GW, Lawrence VA, Cornell JE (2006). Preoperative Pulmonary Risk Stratification for Noncardiothoracic Surgery Systematic Review for the American College of Physicians. Ann Intern Med.

[8] McAlister FA, Bertsch K, Man J, Bradley J, Jacka M (2005). Incidence of and Risk Factors for Pulmonary Complications after Nonthoracic Surgery. Am J Respir Crit Care Med.

[9] Miskovic A, Lumb AB (2017). Postoperative pulmonary complications. Br J Anaesth.

[10] McAlister FA, Khan NA, Straus SE, Papaioakim M, Fisher BW, Majumdar SR (2003). Accuracy of the Preoperative Assessment in Predicting Pulmonary Risk after Nonthoracic Surgery. Am J Respir Crit Care Med.

[11] Qaseem A, Snow V, Fitterman N, Hornbake ER, Lawrence VA, Smetana GW (2006). Risk Assessment for and Strategies To Reduce Perioperative Pulmonary Complications for Patients Undergoing Noncardiothoracic Surgery A Guideline from the American College of Physicians. Ann Intern Med.

[12] De Oliveira GS, McCarthy RJ, Davignon K, Chen H, Panaro H, Cioffi WG (2017). Predictors of 30-Day Pulmonary Complications after Outpatient Surgery Relative Importance of Body Mass Index Weight Classifications in Risk Assessment. J Am Coll Surg.

[13] Hua M, Brady JE, Li G (2012). A Scoring System to Predict Unplanned Intubation in Patients Having Undergone Major Surgical Procedures. Anesth Analg.

[14] Jeong B-H, Shin B, Eom JS, Yoo H, Song W, Han S (2014). Development of a Prediction Rule for Estimating Postoperative Pulmonary Complications. PLoS ONE.

[15] Gupta H, Gupta PK, Schuller D, Fang X, Miller WJ, Modrykamien A (2013). Development and Validation of a Risk Calculator for Predicting Postoperative Pneumonia. Mayo Clin Proc.

[16] S A, da Costa LGV, Hemmes SNT, Canet J, Hedenstierna G, Jaber S (2018). The LAS VEGAS risk score for prediction of postoperative pulmonary complications An observational study. Eur J Anaesthesiol.

[17] Foster CA, Charles EJ, Turrentine FE, Sohn M-W, Kron IL, Jones RS (2019). Development and Validation of Procedure-Specific Risk Score for Predicting Postoperative Pulmonary Complication A NSQIP Analysis. J Am Coll Surg.

[18] Arozullah AM, Daley J, Henderson WG, Khuri SF (2000). Multifactorial Risk Index for Predicting Postoperative Respiratory Failure in Men After Major Noncardiac Surgery. Ann Surg.

[19] Arozullah AM, Khuri SF, Henderson WG, Daley J (2001). Development and Validation of a Multifactorial Risk Index for Predicting Postoperative Pneumonia after Major Noncardiac Surgery. Ann Intern Med.

[20] Canet J, Gallart L, Gomar C, Paluzie G, Vallès J, Castillo J (2010). Prediction of Postoperative Pulmonary Complications in a Population-based Surgical Cohort. Anesthesiology.

[21] Ozrazgat-Baslanti T, Thottakkara P, Huber M, Berg K, Gravenstein N, Tighe P (2016). Acute and Chronic Kidney Disease and Cardiovascular Mortality After Major Surgery Ann. Surg.

[22] Prowle JR, Kam EPY, Ahmad T, Smith NCE, Protopapa K, Pearse RM (2016). Preoperative renal dysfunction and mortality after non-cardiac surgery Preoperative renal dysfunction and mortality after non-cardiac surgery. Br J Surg.

[23] Gajdos C, Hawn MT, Kile D, Robinson TN, Henderson WG (2013). Risk of Major Nonemergent Inpatient General Surgical Procedures in Patients on Long-term Dialysis. JAMA Surg.

[24] Kheterpal S, Shanks AM (2007). Predictors of Postoperative Acute Renal Failure after Noncardiac Surgery in Patients with Previously Normal Renal Function. Anesthesiology.

[25] Romagnoli S, Zagli G, Tuccinardi G, Tofani L, Chelazzi C, Villa G (2016). Postoperative acute kidney injury in high-risk patients undergoing major abdominal surgery. J Crit Care.

[26] Grams ME, Sang Y, Coresh J, Ballew S, Matsushita K, Molnar MZ (2016). Acute Kidney Injury After Major Surgery A Retrospective Analysis of Veterans Health Administration Data. Am J Kidney Dis.

[27] Myles PS, Bellomo R, Corcoran T, Forbes A, Peyton P, Story D (2018). Restrictive versus Liberal Fluid Therapy for Major Abdominal Surgery. N Engl J Med.

[28] Mizota T, Yamamoto Y, Hamada M, Matsukawa S, Shimizu S, Kai S (2017). Intraoperative oliguria predicts acute kidney injury after major abdominal surgery. Br J Anaesth.

[29] Kheterpal S, Tremper KK, Heung M, Rosenberg AL, Englesbe M, Shanks AM (2009). Development and Validation of an Acute Kidney Injury Risk Index for Patients Undergoing General Surgery. Anesthesiology.

[30] Hobson C, Ozrazgat-Baslanti T, Kuxhausen A, Thottakkara P, Efron PA, Moore FA (2015). Cost and Mortality Associated With Postoperative Acute Kidney Injury Ann. Surg.

[31] Park S, Cho H, Park S, Lee S, Kim K, Yoon HJ (2019). Simple Postoperative AKI Risk (SPARK) Classification before Noncardiac Surgery A Prediction Index Development Study with External Validation. JASN.

[32] Eker HH, van Ramshorst GH, de Goede B, Tilanus HW, Metselaar HJ, de Man RA (2011). A prospective study on elective umbilical hernia repair in patients with liver cirrhosis and ascites. Surgery.

[33] Neeff H, Mariaskin D, Spangenberg H-C, Hopt UT, Makowiec F (2011). Perioperative Mortality After Non-hepatic General Surgery in Patients with Liver Cirrhosis an Analysis of 138 Operations in the 2000s Using Child and MELD Scores. J Gastrointest Surg.

[34] Bloch RS (1985). Cholecystectomy in Patients With Cirrhosis A Surgical Challenge. Arch Surg.

[35] Ziser A, Plevak DJ, Wiesner RH, Rakela J, Offord KP, Brown DL (1999). Morbidity and mortality in cirrhotic patients undergoing anesthesia and surgery. Anesthesiology.

[36] Teh SH, Nagorney DM, Stevens SR, Offord KP, Therneau TM, Plevak DJ (2007). Risk Factors for Mortality After Surgery in Patients With Cirrhosis. Gastroenterology.

[37] Mansour A, Watson W, Shayani V, Pickleman J (1997). Abdominal operations in patients with cirrhosis Still a major surgical challenge. Surgery.

[38] Telem DA, Schiano T, Goldstone R, Han DK, Buch KE, Chin EH (2010). Factors That Predict Outcome of Abdominal Operations in Patients With Advanced Cirrhosis. Clin Gastroenterol Hepatol.

[39] Farnsworth N, Fagan SP, Berger DH, Awad SS (2004). Child-Turcotte-Pugh versus MELD score as a predictor of outcome after elective and emergent surgery in cirrhotic patients. Am J Surg.

[40] Perkins L, Jeffries M, Patel T (2004). Utility of preoperative scores for predicting morbidity after cholecystectomy in patients with cirrhosis. Clin Gastroenterol Hepatol.

[41] Runyon BA (1986). Surgical procedures are well tolerated by patients with asymptomatic chronic hepatitis. J Clin Gastroenterol.

[42] Hickman L, Tanner L, Christein J, Vickers S (2019). Non-Hepatic Abdominal Surgery in Patients with Cirrhotic Liver Disease. J Gastrointest Surg.

[43] Friedman LS (1999). The risk of surgery in patients with liver disease. Hepatology.

[44] O'Leary JG, Yachimski PS, Friedman LS (2009). Surgery in the Patient with Liver Disease. Trans Am Clin Climatol Assoc.

[45] Patel T (1999). Surgery in the Patient With Liver Disease. Mayo Clin Proc.

[46] Pugh RNH, Murray-Lyon IM, Dawson JL, Pietroni MC, Williams R (1973). Transection of the oesophagus for bleeding oesophageal varices. Br J Surg.

[47] Malinchoc M, Kamath PS, Gordon FD, Peine CJ, Rank J, ter Borg PCJ (2000). A model to predict poor survival in patients undergoing transjugular intrahepatic portosystemic shunts. Hepatology.

[48] Northup PG, Friedman LS, Kamath PS (2019). AGA Clinical Practice Update on Surgical Risk Assessment and Perioperative Management in Cirrhosis Expert Review. Clin Gastroenterol Hepatol.

[49] Chee YL, Crawford JC, Watson HG, Greaves M (2008). Guidelines on the assessment of bleeding risk prior to surgery or invasive procedures British Committee for Standards in Haematology. Br J Haematol.

[50] Lindblad B, Eriksson A, Bergqvist D (1991). Autopsy-verified pulmonary embolism in a surgical department Analysis of the period from 1951 to 1988. Br J Surg.

[51] Heit JA (2015). Epidemiology of venous thromboembolism. Nat Rev Cardiol.

[52] Ramanathan R, Lee N, Duane TM, Gu Z, Nguyen N, Potter T (2016). Correlation of venous thromboembolism prophylaxis and electronic medical record alerts with incidence among surgical patients. Surgery.

[53] Sandler DA, Martin JF (1989). Autopsy Proven Pulmonary Embolism in Hospital Patients Are We Detecting Enough Deep Vein Thrombosis?. J R Soc Med.

[54] Stein PD, Henry JW (1995). Prevalence of Acute Pulmonary Embolism Among Patients in a General Hospital and at Autopsy. Chest.

[55] Kim JYS, Khavanin N, Rambachan A, McCarthy RJ, Mlodinow AS, De Oliveria GS (2015). Surgical Duration and Risk of Venous Thromboembolism. JAMA Surg.

[56] Anderson FA (2003). Risk Factors for Venous Thromboembolism. Circulation.

[57] White R, Zhou H, Romano P (2003). Incidence of symptomatic venous thromboembolism after different elective or urgent surgical procedures. Thromb Haemost.

[58] Gould MK, Garcia DA, Wren SM, Karanicolas PJ, Arcelus JI, Heit JA (2012). Prevention of VTE in Nonorthopedic Surgical Patients. Chest.

[59] Caprini JA (2005). Thrombosis Risk Assessment as a Guide to Quality Patient Care. Dis Mon.

[60] Bahl V, Hu HM, Henke PK, Wakefield TW, Campbell DA, Caprini JA (2010). A Validation Study of a Retrospective Venous Thromboembolism Risk Scoring Method Ann. Surg.

[61] Pannucci CJ, Bailey SH, Dreszer G, Fisher Wachtman C, Zumsteg JW, Jaber RM (2011). Validation of the Caprini Risk Assessment Model in Plastic and Reconstructive Surgery Patients. J Am Coll Surg.

[62] Obi AT, Pannucci CJ, Nackashi A, Abdullah N, Alvarez R, Bahl V (2015). Validation of the Caprini Venous Thromboembolism Risk Assessment Model in Critically Ill Surgical Patients. JAMA Surg.

[63] Pannucci CJ, Swistun L, MacDonald JK, Henke PK, Brooke BS (2017). Individualized Venous Thromboembolism Risk Stratification Using the 2005 Caprini Score to Identify the Benefits and Harms of Chemoprophylaxis in Surgical Patients A Meta-analysis. Ann Surg.

[64] Rogers SO, Kilaru RK, Hosokawa P, Henderson WG, Zinner MJ, Khuri SF (2007). Multivariable Predictors of Postoperative Venous Thromboembolic Events after General and Vascular Surgery Results from the Patient Safety in Surgery Study. J Am Coll Surg.

[65] Malone DL, Genuit T, Tracy JK, Gannon C, Napolitano LM (2002). Surgical Site Infections Reanalysis of Risk Factors. J Surg Res.

[66] Kaye KS, Schmit K, Pieper C, Sloane R, Caughlan KF, Sexton DJ (2005). The Effect of Increasing Age on the Risk of Surgical Site Infection. J Infect Dis.

[67] van Walraven C, Musselman R (2013). The Surgical Site Infection Risk Score (SSIRS) A Model to Predict the Risk of Surgical Site Infections. McBryde ES, organizador. PLoS ONE.

[68] Classen DC, Evans RS, Pestotnik SL, Horn SD, Menlove RL, Burke JP (1992). The timing of prophylactic administration of antibiotics and the risk of surgical-wound infection. N Engl J Med.

[69] Hawn MT, Richman JS, Vick CC, Deierhoi RJ, Graham LA, Henderson WG (2013). Timing of Surgical Antibiotic Prophylaxis and the Risk of Surgical Site Infection. JAMA Surg.

[70] de Jonge SW, Gans SL, Atema JJ, Solomkin JS, Dellinger PE, Boermeester MA (2017). Timing of preoperative antibiotic prophylaxis in 54,552 patients and the risk of surgical site infection A systematic review and meta-analysis. Medicine.

[71] Muilwijk J, Hof S van den, Wille JC (2007). Associations Between Surgical Site Infection Risk and Hospital Operation Volume and Surgeon Operation Volume Among Hospitals in the Dutch Nosocomial Infection Surveillance Network. Infect Control Hosp Epidemiol.

[72] Curcio D, Cane A, Fernández F, Correa J (2019). Surgical site infection in elective clean and clean-contaminated surgeries in developing countries. International Journal of Infectious Diseases.

[73] Culver DH, Horan TC, Gaynes RP, Martone WJ, Jarvis WR, Emori TG (1991). Surgical wound infection rates by wound class, operative procedure, and patient risk index. Am J Med.

[74] Haley RW, Culver DH, Morgan WM, White JW, Emori TG, Hooton TM (1985). Identifying patients at high risk of surgical wound infection. Am J Epidemiol.

